# Does the Vaccination against Tick-Borne Encephalitis Offer Good Value for Money for Incidence Rates below the WHO Threshold for Endemicity? A Case Study for Germany

**DOI:** 10.3390/vaccines12101165

**Published:** 2024-10-12

**Authors:** Malina Müller, Hannah Lintener, Vivien Henkel, Andreas Pilz, Kate Halsby, Claudius Malerczyk, Harish Madhava, Jennifer C. Moïsi, Holly Yu, Katharina Schley

**Affiliations:** 1 WifOR Institute, 64283 Darmstadt, Germany; 2 Division of Public Health, Social and Preventive Medicine, Center for Preventive Medicine and Digital Health, Medical Faculty Mannheim, Heidelberg University, 68167 Mannheim, Germany; 3 Pfizer Corporation Austria GmbH, 1210 Vienna, Austria; andreas.pilz@pfizer.com; 4 Pfizer Ltd., Tadworth KT20 7 NY, UK; kate.halsby@pfizer.com (K.H.); harish.madhava@pfizer.com (H.M.); 5 Pfizer Pharma GmbH, 10117 Berlin, Germany; claudius.malerczyk@pfizer.com (C.M.); katharina.schley@pfizer.com (K.S.); 6 Pfizer, 75014 Paris, France; jennifer.moisi@pfizer.com; 7 Pfizer Inc., Collegeville, PA 19426, USA; holly.yu@pfizer.com

**Keywords:** tick-borne encephalitis, cost-effectiveness analysis, incidence threshold analysis, infectious disease modeling, comparative analysis, tick-borne diseases

## Abstract

Tick-borne encephalitis (TBE) is a viral infection affecting the central nervous system (CNS) with potential long-term consequences including neurological sequelae. Vaccination is critical to reduce TBE morbidity and mortality, as no antiviral treatment is available. The World Health Organization (WHO) defines areas with an incidence of ≥5 cases/100,000 PPY as highly endemic and recommends that vaccination is offered to all individuals in these areas. However, access to TBE vaccination depends on recommendations and funding by national or subnational decision-makers. We assessed if TBE vaccination could offer good value for money at incidences below this threshold. Methods: A closed-cohort Markov model was developed to estimate the cost-effectiveness of TBE vaccination. We compared primary vaccination applied to the whole population (aged above 1 year) and to a subpopulation aged between 60 and 85 years to a scenario without vaccination. Since TBE incidence is often underestimated, we included under-ascertained TBE cases and non-CNS TBE infections. Germany was used as a case study due to the availability of detailed incidence data. Results: Our incidence threshold analysis showed that TBE vaccination offers good value for money well below the WHO threshold in most of the analyzed scenarios. Conclusions: Our results support a recommendation for TBE vaccination even in settings with low numbers of reported cases, especially for older patients. Furthermore, this analysis identified major research gaps regarding the costs, utilities, and clinical progression of TBE.

## 1. Introduction

Tick-borne encephalitis (TBE) is an arboviral infection of the genus flavivirus and is endemic in regions of Europe and Asia [1]. Global warming and other environmental and social factors are changing the interaction of humans and vectors, increasing the risk of vector-borne diseases [2,3]. The incidence of TBE in humans has risen significantly over the past decades, and endemic regions have expanded [2]. Thus, TBE has become a growing challenge for European healthcare systems [4].

Among individuals exposed to TBE virus (TBEv), 70–98% experience only subclinical infection, which is rarely detected. Of those with clinical symptoms, a small proportion develop an abortive form of TBE disease, limited to nonspecific symptoms including fever and headache, whereas others develop CNS disease, with a severe presentation such as meningitis or meningoencephalomyelitis, of which an estimated 35–58% develop long-term neurological sequelae [5,6]. The relative burden of non-CNS vs. CNS disease is poorly understood, as few non-CNS cases are detected by standard-of-care testing. In addition, CNS disease may also be underdiagnosed. For instance, a recent analysis from Germany suggests that hospitalized patients with CNS symptoms potentially related to TBE are often not tested for TBE [7].

As there is no available antiviral treatment, preventive interventions, including vaccination, are needed to reduce the morbidity and mortality due to TBE [8]. In Europe, two inactivated TBE vaccines are available for adults: FSME-Immun^®^ (Pfizer), in some countries distributed as Ticovac^®^, and Encepur^®^ (Bavarian Nordic). Both vaccines are approved for adults as well as for children above one year of age and show good safety and immunogenicity, as well as effectiveness in real-world use [9,10]. The World Health Organization has recommended offering TBE vaccination to all age groups in areas that are highly endemic for TBE (mean pre-vaccination incidence TBE ≥ 5/100,000 PPY) [11].

Generally, a primary immunization series of three doses is needed, with a first booster after three years and a subsequent booster every three to five years, depending on age. However, recent estimates of vaccine effectiveness (VE) suggest a longer duration of protection, likely allowing for prolonged booster intervals of 10 years [10,12].

Based on an online survey on the uptake of TBE vaccination among household members including adults and children, TBE vaccine uptake (defined as receipt of at least one dose of TBE vaccine) was highly variable across European countries, ranging from 7 to 81% in endemic countries and from 1 to 8% in non-endemic countries [13].

Access to TBE vaccination heavily depends on vaccine recommendation and reimbursement. These decisions are made at a national or subnational level. With the increasing public health threat posed by TBE, further evidence, particularly on the economic value of expanding vaccination programs, is vital to support health policy and financing decisions [14,15]. Several previously published studies of the cost-effectiveness of TBE vaccination in Sweden [4,16], Estonia [17], and Slovenia [14,18], as well as among French troops deployed to Kosovo [19], all identified incidence as the primary driver of cost-effectiveness [16,20]. It is of great interest to link TBE incidence with cost-effectiveness and assess if the WHO incidence threshold of 5/100,000 PPY or rates lower than that can be value for money to support funding.

In our analysis, we determine the TBE incidence rate at which vaccination against TBE is good value for money among two target populations: the whole population (one year of age and above) or a subset of the population, i.e., individuals aged 60–85 years, using Germany as a case study. Given the high degree of uncertainty around the published TBE incidence data as well as other data limitations, the analysis explores different model scenarios, including accounting for the under-ascertainment of CNS infections through existing surveillance, the health and economic burden of non-CNS infections, the uptake of primary vaccination, the yearly waning rate for vaccine effectiveness, and the discount rate of health utilities.

## 2. Materials and Methods

### 2.1. General Model Settings

We used Microsoft Excel (Version 2409 Build 16.0.18025.20030) to develop a cost-effectiveness model that is adjustable to various European countries and customizable for different vaccination strategies, incidence levels, and under-ascertainment of TBE in general and non-CNS TBEv infections.

The model follows a closed-cohort approach with age groups from 1 year to 85 years, with cycle lengths of 1 year, overall simulating a period of 85 years. The model considers only direct costs from a payer’s perspective.

We analyzed two vaccination strategies. Strategy 1 covers primary vaccination (3 doses within one year) for the whole population and strategy 2 covers primary vaccination in the population aged ≥60 years. Both strategies were compared against a scenario without vaccination.

### 2.2. Model Structure

In the model, mutually exclusive Markov health states were defined based on the natural course of TBEv infections [21,22]. Table 1 provides an overview of health states and their respective definitions. Individuals enter the model as susceptible, defined by the absence of a current TBEv infection. All TBEv infections are assumed to be associated with the European TBE subtype (TBEv-EU), the most prevalent TBE subtype in Europe [23]. Following the TBE disease classification described in Mickienné et al. (2002) [21], we categorized neurological manifestations of TBE into three health states: TBE 1, characterized by primarily meningeal symptoms, including fever, headache, neck rigidity, and nausea; TBE 2, a disease with monofocal symptoms of the CNS and/or moderate diffuse brain dysfunction; TBE 3, a disease with multifocal CNS symptoms and/or severe diffuse brain dysfunction. A small fraction of patients infected with TBE develop a febrile or abortive form of infection, characterized by nonspecific symptoms in the form of a febrile illness with a headache without involvement of the CNS [6]. To account for these non-CNS TBE cases, we added health states for TBEv infections without CNS involvement, with a distinction between non-CNS TBE cases in inpatient and outpatient settings in the model. Similar to previous CE models for TBE vaccination [14,17,18], we classified sequelae health states according to Bohr et al. (1998) [24] into mild, moderate, and severe sequelae.

Based on the risk of infection, estimated from the average incidence of TBE in Germany in the years 2018–2022 [25], individuals move from the susceptible state to TBEv infection, then may progress to one of three neurological manifestations of TBE (TBE 1, 2, or 3) requiring hospitalization. Hospitalized TBEv cases without CNS involvement in Germany are included in the total number of TBE cases published by the Robert Koch Institute (RKI). As German authorities do not stratify the case numbers by patients with CNS involvement and non-CNS cases, the proportion of these non-CNS cases as a percentage of the total is assumed.

**Figure 1:**
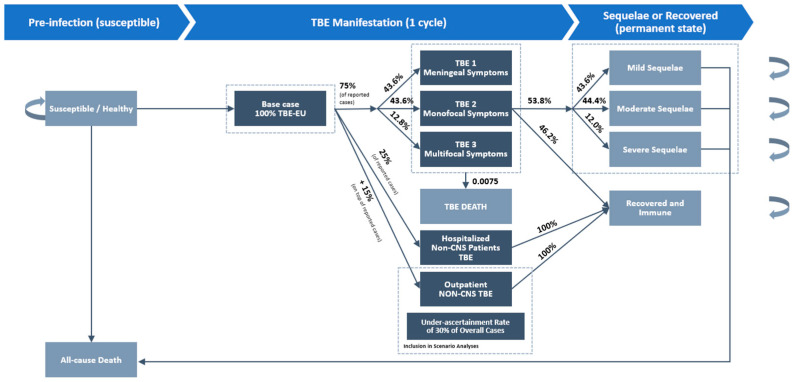
Stylized depiction of the Markov model.

Thus, in the base case, we assume that 75% of all reported TBE cases in Germany are hospitalized TBEv infections with CNS involvement, whereas 25% of cases are assumed to be associated with hospitalized TBEv infection without CNS involvement [26] (Figure 1). Moreover, we explore the impact of including non-CNS cases in the outpatient setting.

We derived the transition rates for hospitalized TBE cases with CNS involvement and sequelae of TBE from Mickienné et al. (2002) [21], which reports data from a sample of 133 consecutively admitted patients with TBE with CNS involvement in Lithuania who were treated for one year [21]. In the base case, the same transition rates of Mickiené et al. (2002) are applied to all patients, including children and adolescents, as the data on disease progression in children are rare [16,17,18]. All patients remain in one of the disease manifestation states for one cycle (one year). Based on Mickiene et al. (2002), we assume that in 43.6% of all hospitalized TBE cases with CNS involvement, the infection manifests as TBE 1, whereas 43.6% develop TBE 2, and 12.8% develop TBE 3 [21].

Non-CNS TBEv cases do not progress to sequelae states in the next cycle but move to “recovered and immune” for the remainder of the model. Of the patients with CNS involvement (TBE 1–3), 53.8% of all patients hospitalized for TBE with CNS involvement move to one of three sequelae states while 46.2% of hospitalized CNS patients move to “recovered immune”. Of these patients with follow-up consequences, 43.6% move to mild sequelae, 44.4% to moderate sequelae, and 12.0% progress to severe sequelae [21]. The transition probabilities are the same across all acute disease states. Similar to the approach taken in the previous literature [4,14,17,18], it is assumed that the patients that develop long-term sequelae will suffer the consequences for the remainder of their life.

### 2.3. Base Case Inputs

Table 2 provides an overview of the base case model inputs. While we present a case study for Germany, our model is adaptable to other European countries. For the German base case, we extracted the population figures from Eurostat [27,28] and the age-specific incidence rates for TBE from SURVSTAT of the RKI [25]. To account for annual fluctuations in reported cases, we implemented the mean incidence of TBE for the years 2018–2022 in the model.

### 2.4. Vaccination

We analyzed two vaccination strategies. Strategy 1 includes a three-dose primary vaccination series in the population aged ≥1 year to 85 years and strategy 2 includes only the population aged ≥60 years to 85 years. Rapid immunization schedules and other booster intervals were out-of-scope of this analysis. We compared both strategies against a scenario without vaccination. For both strategies, we assumed an uptake of primary immunization, defined as completion of three primary doses of 19% of the total population against TBE in defined risk areas in 2020 (primary immunization and, if applicable, timely booster vaccination) published by the RKI [29].

The model utilizes VE from Nygren et al. (2022) [10]. In both strategies, we employed a VE of 96.6% for the first three years after primary immunization [10]. There is currently some uncertainty around how VE develops beyond a timeframe of 10 years [10,12,36]; therefore, we assumed a yearly waning rate of 5% after the initial three years in the base case [29] based on findings from other disease areas.

### 2.5. Health Utility (HU) Estimates

Due to a lack of data on the health utilities of patients with TBE, we employed utility values from diseases with a similar clinical presentation. This approach, while requiring several assumptions, was also adopted in previous CE models analyzing TBE vaccination [4,16,17,18,37].

Health utilities (HUs) are cardinal values that reflect an individual’s preferences for different health outcomes, with zero corresponding to death and one to perfect health [38]. We derived health utilities for the neurological manifestations of TBE (TBE1, TBE2, and TBE3) from a publication from the United States on the HUs of *Neisseria meningitidis* serogroup B, a bacterial infection which, similar to TBE, manifests in the form of neurological inflammation. In this publication, HUs are not considered constant over a year but are multiplied by the average length of hospital stay. Following the conservative approach, we applied utilities from meningitis (inpatient care, non-intensive care unit (ICU)) for 5 days to TBE 1. We used utilities for meningitis requiring ICU admission of 2 days followed by a 2-day inpatient stay (post-ICU) to both TBE 2 and TBE 3 [33]. Given the nonspecific and febrile symptoms associated with non-CNS TBEv infection, we assumed influenza to be a close surrogate option for health utilities [6]. Therefore, we used utility values from influenza collected in Spain for inpatient and outpatient non-CNS TBEv states [34]. Based on epidemiological data on the progression of non-CNS TBEv infection [33], an average duration of symptoms of 5 days was assumed and applied, following the utility calculation for TBE 1–3 [33].

Similar to previous TBE CE models [14,16,17,18,39], we extracted the health utilities associated with TBE sequelae from a publication that observed long-term impairment and health-related quality of life in patients with a past infection of *Haemophilus influenzae* type b. HU values were assumed to be 0.98 in mild sequelae, 0.84 in moderate sequelae, and 0.37 in severe sequelae. The HU values for sequelae states were assigned for the whole cycle.

### 2.6. Cost Estimates

Since cost data are particularly scarce for TBE, we only included direct medical costs in the CE model. We selected the cost data reported in Mihajlovic et al. (2019) [18] as the most comprehensive and recent cost estimation for direct costs associated with TBE. The extracted cost estimates were adjusted for country price level differences based on relative price level differences provided by the OECD [40] and inflation. Inflation rates were derived from the purchasing power consumer price index from the German Statistical Office [41]. Germany-specific vaccine costs per dose were extracted with reference costs for FSME Immun, using the pharmacy retail price in the IQWiG Guideline [30,32].

### 2.7. Analysis

To evaluate the incidence threshold at which TBE vaccination would be considered good value for money, we calculated the incremental cost-effectiveness ratio (ICER) for TBE vaccination compared to no vaccination. We defined good value for money as an ICER less than a willingness to pay threshold (WTP). Given that no WTP threshold exists in Germany, we used the commonly accepted WHO WTP definition, which is three times the gross domestic product (GDP) per capita. In Germany, GDP per capita was EUR 35,880 in 2022 and we applied three times to that GDP (WTP threshold 3: 107,640). We further explored lower WTP thresholds such as one times the GDP per capita (WTP threshold 1: EUR 35,880 for Germany) and two times the GDP per capita (WTP threshold 2: EUR 71,760 for Germany) [42,43].

We calculated the incidence threshold for the base case and conducted one-way scenario or sensitivity analysis to account for uncertainty around certain parameters. The scenarios included a lower VE of 93.7% [10], an increase in the uptake of primary immunization to the median value of influenza vaccine uptake in Germany in ≥60-year-olds in the years 2018–2021 [29]. Additionally, we included 15% of outpatient non-CNS cases on top of the reported cases and an under-ascertainment rate of 30% of the overall cases in the scenario analyses. Based on the ongoing discussion around the discounting of health benefits, we implemented a discounting rate of 1.5% to health outcomes in the scenario analyses while keeping the discounting rate for costs at 3% [18,44]. The assumptions for the base case and scenario analyses are described in Table 3.

## 3. Results

### 3.1. Base Case Results

Table 4 displays the results of the base case analysis with an incidence of 0.67 cases/100,000 PPY as observed in Germany for the years 2018–2022 for the whole population and an incidence of 0.77/100,000 PPY for the age group 60–85 years. Strategy 2, targeting only the population between 60 and 85 years results in an ICER of EUR 82,358; therefore, this could be considered good value for money compared to WTP threshold 3 (EUR 107,640). When targeting the whole population, our results show an ICER of EUR 253,529; therefore, strategy 1 would not be considered good value for money at any of the WTP thresholds.

Although the scenario analysis further emphasizes a substantial impact of under-ascertainment of reported cases alongside the discount rate of HU, leading to much lower ICERs, the base case analysis shows that vaccination only offers a good value for money in an older population. Figure S1 in the Supplementary Materials illustrates the impact of varying the input parameters on the ICER.

### 3.2. Incidence Threshold Analysis

**Figure 2:**
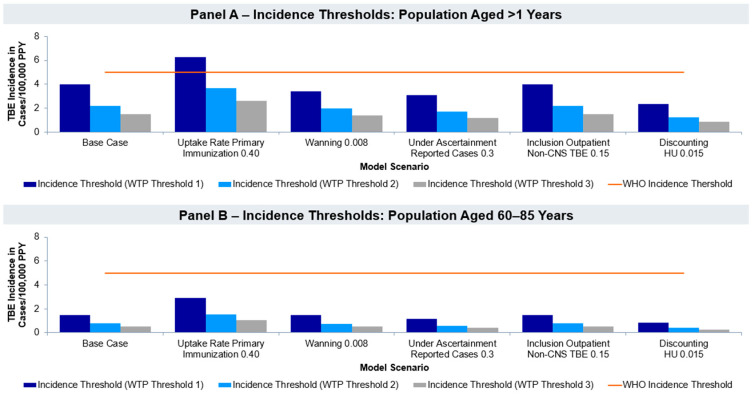
Incidence thresholds for strategy 1 and 2 in different model scenarios. Note: The applied WTP thresholds were EUR 107,640 (3 × GDP per capita/WTP threshold 3), EUR 71,760 (2 × GDP per capita/WTP threshold 2), and EUR 35,880 (1 × GDP per capita/WTP threshold 1) [43].

The results of the base case using the observed incidence rate in Germany of 0.67/100,000 PPY show that primary immunization offers significant value for money when considering a WTP equal to three times the German GDP and targeting the population ≥ 60 to 85 years. Figure 2 illustrates the incidence thresholds for strategies 1 and 2, calculated separately for each of the three WTP thresholds. The figure displays the WHO threshold for endemicity at ≥5 cases/100,000 PPY as an incidence reference value. In the base case, primary immunization of the whole population would offer good value for money at incidence levels of 1.5 per 100,000/PPY for WTP 3, 2.2 per 100,000/PPY for WTP 2, and 4.0 per 100,000/PPY for WTP 1. In a scenario with an increased uptake of 40% of the primary immunization series, vaccination of the general population would offer significant value for money for WTP 3 at an incidence rate of 2.60/100,000 PPY. Including outpatient non-CNS cases does not reduce the incidence threshold considerably compared to the base case. Increasing the proportion of under-ascertainment to 30% of cases decreases the incidence threshold to 1.16/100,000 for WTP 3. Decreasing the rate of discounting of health utilities reduces the incidence below 5/100,000 for all WTP 1, 2, and 3, especially for WTP 3 even below 1/100,000 to 0.84 per 100,000/PPY (Table S1).

For a population aged between 60 and 85 years, the base case resulted in an ICER below WTP 3. In all scenario analyses, vaccination of this age group offered significant value for money at incidence rates between 0.28 and 1 cases per 100,000 PPY for WTP 3 (Table S1).

## 4. Discussion

To our knowledge, this is the first study to analyze the incidence level at which primary immunization against TBE would offer good value for money, based on a range of assumed willingness to pay thresholds. In particular, the model results show that TBE vaccination delivers good value for money already below the endemicity threshold defined by the WHO (an incidence of ≥5 cases/100,000 PPY), particularly when targeting older adults. The ICER results of this study align with the findings of previous publications [4,16,17] stating that vaccination strategies targeted at older age groups generate more favorable ICERs.

This analysis highlights the substantial impact of under-ascertainment of TBE cases on the value of the vaccination. Surveillance data only reports medically attended cases and might therefore be an underestimation of the actual disease burden. Furthermore, not all medically attended TBE cases are diagnosed, even in a clinic setting, as reported in a recent study [7]. Using an underestimated incidence to evaluate cost-effectiveness may lead to suboptimal vaccine recommendations and reimbursement decisions. Our analysis highlights the importance of considering willingness to pay as well as other factors that drive cost-effectiveness to determine the appropriate endemicity thresholds for defining TBE risk areas. Defining these thresholds is critical to public health, as Ghiani et al. recently observed that declaring new risk areas can substantially increase vaccination uptake [45].

In Germany, vaccination is recommended by the German Standing Committee on Vaccination (STIKO) for all people living in, working in, or traveling to risk areas, and is reimbursed for these groups [1]. Risk areas are classified annually by the German Public Health Institute (Robert Koch Institute (RKI)) on a county level (“Stadt- or Landkreis”, i.e., NUTS-3 administrative district) as an area with a five-year incidence above 1/100,000 inhabitants within the district and surrounding districts. This is the outcome of a discussion by a panel of experts in 2007, resulting in the change from a case-based definition to an incidence-based definition and taking into account the average nationwide incidence at that time, as well as in existing risk areas [46].

The presented cost-effectiveness results suggest that vaccination of 60–85-year-olds could provide good value for money at a national level, based on the currently reported incidence. However, no formal WTP threshold exists in Germany and the authors rely on a hypothetical WTP threshold of three times the GDP per capita as defined by the WHO.

The incidence threshold analysis highlights that the vaccination could be a good use of resources at incidences above 1.51/100,000 PPY for the whole German population and 0.52/100,000 for a population between 60 and 85 years of age in the base case. Considering the impact of under-ascertainment, this could be reduced to 1.16/100,000 PPY for the whole German population and 0.40/100,000 for a population between 60 and 85 years of age. However, the model and modeling assumptions are inherently conservative. Additionally, the model evaluated the cost of disease only from a payer perspective and did not consider societal impacts such as productivity losses. For instance, a study by the RKI, monitoring more than 500 TBE patients on recovery and sequelae, reports that 88.4% of cases require sick leave and 10.3% of cases planned or reported a premature retirement due to sequelae [47]. Furthermore, 30.5% of TBE cases reported a negative impact of TBE on their work performance. Both absenteeism and presentism prevalent in TBE cases can cause substantial societal costs which, if taken into consideration, further increase the cost-effectiveness of the TBE vaccination. Accordingly, the incidence thresholds could be significantly lower than what is reported above.

Furthermore, we assumed an under-ascertainment for adults of 30%. However, recent evidence suggests that under-ascertainment in children and adolescents is much higher than previously thought [48]. Additionally, new evidence is emerging which demonstrates that TBE in children is more severe than previously believed and does not only manifest in mainly mild forms. The RKI reports that more than 50% of TBE cases in children (37/66) had a moderate or severe course of disease [49]. Again, this substantiates the conservative nature of this modeling approach. Accordingly, this could further reduce the incidence threshold estimates.

The current designation for risk areas in Germany, and thus for a vaccine recommendation, is below the above reported results. However, in light of the conservative nature of the analysis and that German data was solely used as a case study in this analysis, the absolute values presented above should be cautiously compared with the current RKI definition of risk areas.

A general limitation of this analysis is the lack of robustness of available TBE data, which has been reported in prior estimations of the CE of TBE vaccination [4,14,16,17,19]. Due to relatively low case numbers, sample sizes in clinical trials are small and clinical evidence (long-term outcomes, costs, and health utility estimations) is scarce, particularly in children and adolescents. To model the cost-effectiveness of TBE vaccination, we had to make several conservative assumptions based on limited data, including the assumption that there is no correlation between the severity of manifestation states and subsequent sequelae. Moreover, cost data applicable to the respective health states was only available in an aggregated form from Slovenia. Cost data for the German setting was estimated by adjusting this cost data for country price levels and inflation. Given that most assumptions could be considered conservative, the results might underestimate the true value of TBE vaccination.

Generally, surveillance data only reports medically attended cases and might therefore be an underestimation of the actual disease burden. Furthermore, Schley et al. have highlighted that not all patients with symptoms typical for TBE infection such as CNS and other symptoms are tested for TBE infection [7]. This potentially further adds to the potential underestimation of the actual burden of TBE infections.

While national estimates of cost data and resource consumption are preferable in economic evaluations [50], the only comprehensive evidence of the direct treatment costs for TBE comes from previous CE models. Mostly, publications used cost information from personal communication between the publication authors and health authorities in the respective countries. Moreover, there is significant heterogeneity in the definitions associated with the manifestations of TBEv and its sequelae.

## 5. Conclusions

This study aimed to estimate incidence thresholds at which TBE vaccination would be cost-effective and to determine if TBE vaccination would offer significant value for money for incidence rates below 5 per 100,000 PPY, the threshold recommended by the WHO, using Germany as a case study. The results indicate that TBE vaccination is good value for money even in settings where the incidence falls below the WHO threshold of ≥5 cases/100,000 PPY. Primary immunization targeting the subpopulation between 60 to 85 years reaches a WTP threshold of 1 GDP per capita in all scenarios far below an incidence of ≥5 cases/100,000 PPY. Vaccinating the whole population and considering the WTP threshold of three times the GDP per capita delivers good value for money in most scenarios. Overall, our results show that the uptake of primary immunization, the extent of incidence under-ascertainment, and the inclusion of non-CNS TBEv cases have a considerable impact on the ICER and, thus, on the incidence thresholds that can be used to define vaccination policy.

Our findings provide the first comprehensive assessment of the WHO incidence threshold in the context of TBE cost-effectiveness. Despite the severe complications associated with TBEv, vaccination against TBE still has low uptake due to limited recommendations and reimbursement in many European countries. Our analysis shows that TBE vaccination offers significant value for money for settings with lower endemicity and incidence rates below the 5 per 100,000 PPY currently recommended by the WHO. For Germany, the incidence threshold analysis revealed incidence rates above the current incidence rate used for the designation of TBE risk areas in Germany. However, the modeling assumptions are conservative and only consider a payer perspective and thus very likely overestimate the incidence threshold and therefore undervalue the benefit of TBE vaccination. Accordingly, the absolute values above should be cautiously interpreted for Germany.

Improved TBE surveillance is needed to strengthen our understanding of disease burden, derive more robust cost-effectiveness estimates, and increase access to and uptake of TBE vaccines in endemic areas in Europe.

## Figures and Tables

**Table 1:** Definition of TBEv infection health states.

Health State	Definition	Source
Susceptible	No present TBE infection.	
TBE 1	Primarily meningeal symptoms including fever, headache, rigidity of the neck, and nausea.	[21,22]
TBE 2	Disease with monofocal symptoms of the CNS and/or moderate diffuse brain dysfunction.	[21,22]
TBE 3	Disease with multifocal symptoms of the CNS and/or severe diffuse brain dysfunction.	[21,22]
Inpatient non-CNS TBEv	TBEv cases without CNS manifestation in inpatient care. These infections are usually accompanied by unspecific, flu-like symptoms.	[6,21]
Outpatient non-CNS TBEv	TBEv cases without CNS manifestation in outpatient care. These infections are usually accompanied by unspecific, flu-like symptoms.	[6]
TBE death	Death due to TBE	[4,15,17,18]
Mild sequelae	Presence of one or more mild symptoms, including dizziness, memory deficits, headache, tiredness, slight hearing impairment, minor psychological problems, or unsteady gait. Daily life and working abilities are not markedly affected.	[24]
Moderate sequelae	Presence of many or more severe symptoms, ataxia of gait, paresis of the extremities, pronounced dementia, or severe deafness. Patient affected in daily life and working ability.	[24]
Severe sequelae	More pronounced clinical disabilities, often seriously affecting social life and working capabilities, and in a few cases, requiring institutional care.	[24]
Recovered and immune	Recovered from TBE without any sequelae. Immunity persists for the remainder of the model.	
All-cause death	All-cause death, based on age- and gender-stratified data extracted from national life and death tables.	

**Table 2:** Overview of base case model assumptions.

Input Parameter	Base Case Value	Reference
**Population**		
Population by age and gender 2022	Age- and gender-specific	[28]
**Epidemiology**		
Age-specific incidence rate—average from 2018 to 2022	Age- and gender-specific	[25]
**Uptake**		
Proportion of people receiving primary immunization: completion of three doses	0.19	[29]
**Vaccine effectiveness**		
VE for first three years	0.966	[10]
Annual waning rate starting in year four	0.05	Expert assumption
**Transition rates**		
Probability of TBE death	0.008	[21]
Probability of all-cause death—age-specific lifetables 2021	Age- and gender-specific	[27]
Proportion of patients suffering from non-CNS TBEv (inpatient setting), among reported cases	0.25	[21]
Additional non-CNS TBEv (outpatient setting), as a proportion of reported cases	0.15	Expert assumption
Probability of TBE 1	0.436	[21]
Probability of TBE 2	0.436	[21]
Probability of TBE 3	0.128	[21]
Probability of developing lifelong sequelae (Sequelae were classified as “mild”, “moderate”, or “severe”, depending on their influence on the patient’s quality of life, following [24]).	0.538	[21]
Mild sequelae	0.436	[21]
Moderate sequelae	0.444	[21]
Severe sequelae	0.120	[21]
**Cost parameter** Country-adjusted cost value (value in original publication)		
Cost of vaccination (per dose)	EUR 50.12	[30]
Administration costs	EUR 8.62 (EUR 7.90)	[20]
Direct medical annual costs per TBE 1 case	EUR 1627.58 (EUR 1235.00)	[18]
Direct medical annual costs per TBE 2 case	EUR 3841.62 (EUR 2915.00)	[18]
Direct medical annual costs per TBE 3 case	EUR 14,628.48 (EUR 11,100.00)	[18]
Direct medical annual costs, mild sequelae	EUR 98.69 (EUR 70.00)	[18]
Direct medical annual costs, moderate sequelae	EUR 172.00 (EUR 122.00)	[18]
Direct medical annual costs, severe sequelae	EUR 41,589.27 (EUR 28,952.00)	[18]
Direct medical costs, non-CNS TBE (inpatient setting)	EUR 2229.81 (EUR 2033.00)	[31]
Direct medical costs, non-CNS TBE (outpatient setting)	EUR 284.90 (EUR 259.75)	[31]
**Discounting**		
Discount rate (costs)	0.030	[32]
Discount rate (health utility)	0.030	[32]
**Utility values**		
Utility, TBE 1	0.39 × 0.0137 years (duration of 5 days)	[33]
Utility, TBE 2	0.24 × 0.0055 years + 0.28 × 0.0137 years (duration of 7 days)	[33]
Utility, TBE 3	0.24 × 0.0055 years + 0.28 × 0.0137 years (duration of 7 days)	[33]
Utility, non-CNS TBE (inpatient setting)	0.495 × 0.0137 years (duration of 5 days)	[34]
Utility, non-CNS TBE (outpatient setting)	0.495 × 0.0137 years (duration of 5 days)	[34]
Utility, mild sequelae	0.023	[35]
Utility, moderate sequelae	0.160	[35]
Utility, severe sequelae	0.629	[35]

**Table 3:** Base case assumptions and scenario analysis for vaccination strategies.

	Base Case Assumptions Strategies 1 + 2	Variation in Scenario Analysis
Uptake of primary vaccination	0.19	0.40
Yearly waning rate	0.05	0.008
Inclusion of outpatient non-CNS cases	0	0.15
Multiplier to account for under-ascertainment	No	0.3
Discount rate HU	0.03	0.015

**Table 4:** Results in the base case and scenario analyses.

Vaccination Strategy	Vaccination Strategy 1	Vaccination Strategy 2
Target group	Population of ≥1–85 years	Population of ≥60–85 years
Base case averted TBE cases (hospitalized, CNS involvement)	1842	310
Base case gained QALYs	10,318	9125
Base case cost per QALY gained in EUR	EUR 253,529	EUR 82,358
VE for first three years 0.937	EUR 254,891	EUR 82,499
Uptake rate primary immunization 0.40	EUR 459,805	EUR 167,155
Waning 0.008	EUR 230,970	EUR 81,475
Under ascertainment 0.3	EUR 193,144	EUR 62,918
Inclusion of non-CNS TBEv cases (outpatient setting)/rate 0.15	EUR 253,502	EUR 82,355
Discounting HU 0.015	EUR 136,337	EUR 43,981

## Data Availability

Data are however available from the authors upon reasonable request.

## Supplementary Materials

The following supporting information can be downloaded at: https://www.mdpi.com/article/10.3390/vaccines12101165/s1; Figure S1: ICER in the base case scenarios and under variation of other influential parameters using the real-world incidence. Table S1: Incidence thresholds for Strategy 1 and 2 in different model scenarios using the real-world incidence.
