# Correction: Müller et al. Does the Vaccination Against Tick-Borne Encephalitis Offer Good Value for Money for Incidence Rates Below the WHO Threshold for Endemicity? A Case Study for Germany. *Vaccines* 2024, *12*, 1165

**DOI:** 10.3390/vaccines14030254

**Published:** 2026-03-10

**Authors:** Malina Müller, Hannah Lintener, Vivien Henkel, Andreas Pilz, Kate Halsby, Claudius Malerczyk, Harish Madhava, Jennifer C. Moïsi, Holly Yu, Katharina Schley

**Affiliations:** 1 WifOR Institute, 64283 Darmstadt, Germany; hannah.lintener@web.de (H.L.); vivien_henkel@web.de (V.H.); 2 Division of Public Health, Social and Preventive Medicine, Center for Preventive Medicine and Digital Health, Medical Faculty Mannheim, Heidelberg University, 68167 Mannheim, Germany; 3 Pfizer Corporation Austria GmbH, 1210 Vienna, Austria; andreas.pilz@pfizer.com; 4 Pfizer Ltd., Tadworth KT20 7 NY, UK; kate.halsby@pfizer.com (K.H.); harish.madhava@pfizer.com (H.M.); 5 Pfizer Pharma GmbH, 10117 Berlin, Germany; claudius.malerczyk@pfizer.com (C.M.); katharina.schley@pfizer.com (K.S.); 6 Pfizer, 75014 Paris, France; jennifer.moisi@pfizer.com; 7 Pfizer Inc., Collegeville, PA 19426, USA; holly.yu@pfizer.com

The authors would like to make the following corrections to this published paper [1].

In the original publication [1], Mihajlović et al. (2019) [18] was cited as the source for “Direct medical annual costs for mild, moderate, and severe sequelae” in Table 2, “Overview of base case model assumptions”, in line with the citation provided in their manuscript. These cost estimates were originally derived from Šmit (2012) [14]. The table has been corrected to directly cite the original source. In addition, we inadvertently reported disutility values instead of utility values for mild, moderate, and severe sequelae in Table 2. The updated Table 2 is displayed below:

Reference Mihajlović et al. (2019) [18] was cited inappropriately. The citation has now been removed from the following sentences.

Section 1, “Introduction”, sixth paragraph:

“Several previously published studies of the cost-effectiveness of TBE vaccination in Sweden [4,16], Estonia [17], and Slovenia [14], as well as among French troops deployed to Kosovo [18], all identified incidence as the primary driver of cost-effectiveness [16,19].”

Section 2.2. “Model Structure”, first paragraph:

“Similar to previous CE models for TBE vaccination [14,17], we classified sequelae health states according to Bohr et al. (1998) [23] into mild, moderate, and severe sequelae.”

Section 2.5. “Health Utility (HU) Estimates”:

In the first paragraph, “This approach, while requiring several assumptions, was also adopted in previous CE models analyzing TBE vaccination [4,16,17,37].”

In the last paragraph, “Similar to previous TBE CE models [14,16,17,39], we extracted the health utilities associated with TBE sequelae from a publication that observed long-term impairment and health-related quality of life in patients with a past infection of *Haemophilus influenzae* type b [35].” In the same sentence, Livartowski et al. (1996) [35] has now been cited and added at the end of the sentence.

Meanwhile, with the removal of Reference [18] from the above sentences, References [19–24] in the original publication have been resequenced as References [18–23], while the original Reference [18] has been resequenced as Reference [24], in both the main text and Tables 1 and 2.

To prevent any misunderstandings and ensure clarity, the authors would like to change the first sentence in Section 2.2. “Model Structure”:

“In the model, mutually exclusive Markov health states were defined based on the natural course of TBEv infections [21,22].”

into

“Building on previous cost-effectiveness models for TBE vaccination [4,14,16,17] and aligning with the natural course of the disease, we developed a Markov model reflecting a susceptible–infected–recovered/sequelae pathway, with mutually exclusive health states based on TBE progression, while also reviewing and adapting elements from existing models to ensure relevance to our study context [20,21]”.

Reference Šmit et al. (2012) [14] was not cited in the following Section 2.6. “Cost Estimates”:

“Since cost data are particularly scarce for TBE, we only included direct medical costs in the CE model. We selected the cost data reported in Mihajlovic et al. (2019) [18] as the most comprehensive and recent cost estimation for direct costs associated with TBE.”

The citation has now been inserted; the sentences has been rephrased and should read:

“Since cost data are particularly scarce for TBE, we only included direct medical costs in the CE model. We selected the cost data reported in Mihajlovic et al. (2019) [24], which analyzed a combined anti-tick vaccine for Lyme borreliosis and TBE for active TBE manifestations (TBE 1-3) and from Šmit et al. (2012) [14] for mild, moderate and severe sequelae as the most comprehensive and recent cost estimation for direct costs associated with TBE.”

To prevent any misunderstandings and ensure clarity, the authors would like to change the following sentence in Section 2.7. “Analysis”, second paragraph:

“Additionally, we included 15% of outpatient non-CNS cases on top of the reported cases and an under-ascertainment rate of 30% of overall cases in the scenario analyses.”, into

“Additionally, we included 15% of outpatient non-CNS cases on top of the reported cases. Furthermore, an under-ascertainment rate of 30% was applied to overall cases, based on multiple data sources, including surveillance reports and expert assessments of TBE misdiagnosis rates.”

To ensure clarity, the authors would like to add a sentence to Section 4. Discussion, paragraph 6, that now reads now as follows:

“Furthermore, we assumed an under-ascertainment for adults of 30%. However, recent evidence suggests that under-ascertainment in children and adolescents is much higher than previously thought [48]. Additionally, new evidence is emerging which demonstrates that TBE in children is more severe than previously believed and does not only manifest in mainly mild forms. The RKI reports that more than 50% of TBE cases in children (37/66) had a moderate or severe course of disease [49]. Given the estimates published so far for under-ascertainment in Germany and other European countries [7,37,49], we assume this to be a conservative estimate, ensuring that undiagnosed cases and misclassified febrile illnesses are adequately accounted for, strengthening the robustness of our model. Again, this substantiates the conservative nature of this modelling approach. Accordingly, this could further reduce the incidence threshold estimates.”

To ensure clarity, the authors would like to remove non-CNS TBEv cases from the following sentence in Section 5 “Conclusions”, paragraph 1, as their inclusion did not substantially impact the ICER:

“Overall, our results show that the uptake of primary immunization, the extent of incidence under-ascertainment, and the inclusion of non-CNS TBEv cases have a considerable impact on the ICER and, thus, on the incidence thresholds that can be used to define vaccination policy.”

and modify the sentence to:

“Overall, our results show that the uptake of primary immunization and the extent of incidence under-ascertainment have a considerable impact on the ICER and, thus, on the incidence thresholds that can be used to define vaccination policy.”

The authors state that the scientific conclusions are unaffected. This correction was approved by the Academic Editor. The original publication has also been updated.

## Figures and Tables

**Table 2:** Overview of base case model assumptions.

Input Parameter	Base Case Value	Reference
**Population**		
Population by age and gender 2022	Age- and gender-specific	[28]
**Epidemiology**		
Age-specific incidence rate—average from 2018 to 2022	Age- and gender-specific	[25]
**Uptake**		
Proportion of people receiving primary immunization: Completion of three doses	0.19	[29]
**Vaccine effectiveness**		
VE for first three years	0.966	[10]
Annual waning rate starting in year 4	0.05	Expert assumption
**Transition rates**		
Probability of TBE-death	0.008	[20]
Probability of all-cause death—age-specific lifetables 2021	Age- and gender-specific	[27]
Proportion of patients suffering from non-CNS TBEv (inpatient setting), among reported cases	0.25	[20]
Additional non-CNS TBEv (outpatient setting), as a proportion of reported cases	0.15	Expert assumption
Probability of TBE 1	0.436	[20]
Probability of TBE 2	0.436	[20]
Probability of TBE 3	0.128	[20]
Probability of developing lifelong sequelae *	0.538	[20]
Mild sequelae	0.436	[20]
Moderate sequelae	0.444	
Severe sequelae	0.120	[20]
**Cost parameter:** Country-adjusted cost value (value in original publication)		
Cost of vaccination (per dose)	EUR 50.12	[30]
Administration costs	EUR 8.62 (EUR 7.90)	[19]
Direct medical annual costs per TBE 1 case	EUR 1627.58 (EUR 1235.00)	[24]
Direct medical annual costs per TBE 2 case	EUR 3841.62 (EUR 2915.00)	[24]
Direct medical annual costs per TBE 3 case	EUR 14,628.48 (EUR 11,100.00)	[24]
Direct medical annual costs: mild sequelae	EUR 98.69 (EUR 70.00)	[14]
Direct medical annual costs: moderate sequelae	EUR 172.00 (EUR 122.00)	[14]
Direct medical annual costs: severe sequelae	EUR 41,589.27 (EUR 28,952.00)	[14]
Direct medical costs: non-CNS TBE (inpatient setting)	EUR 2229.81 (EUR 2033.00)	[31]
Direct medical costs: non-CNS TBE (outpatient setting)	EUR 284.90 (EUR 259.75)	[31]
**Discounting**		
Discount rate (costs)	0.030	[32]
Discount rate (health utility)	0.030	[32]
**Utility values**		
Utility TBE 1	0.39 × 0.0137 years (duration of 5 days)	[33]
Utility TBE 2	0.24 × 0.0055 years + 0.28 × 0.0137 years (duration of 7 days)	[33]
Utility TBE 3	0.24 × 0.0055 years + 0.28 × 0.0137 years (duration of 7 days)	[33]
Utility non-CNS TBE (inpatient setting)	0.495 × 0.0137 years (duration of 5 days)	[34]
Utility non-CNS TBE (outpatient setting)	0.495 × 0.0137 years (duration of 5 days)	[34]
Utility mild sequelae	0.98	[35]
Utility moderate sequelae	0.84	[35]
Utility severe sequelae	0.37	[35]

* Sequelae were classified as “mild”, “moderate”, or “severe”, depending on their influence on the patient’s quality of life, following [23].
