# Supplementary material for: Does the Vaccination against Tick-Borne Encephalitis Offer Good Value for Money for Incidence Rates below the WHO Threshold for Endemicity? A Case Study for Germany
Source: Vaccines (Basel). 2024 Oct 12;12(10):1165. doi: 10.3390/vaccines12101165 (PMC11512403; doi:10.3390/vaccines12101165)
Supplement: Supplementary file 1 [file vaccines-12-01165-s001.zip › vaccines-3096218-supplementary.pdf]

Figure S1: ICER in the base case scenarios and under variation of other influential parameters using the real-world incidence

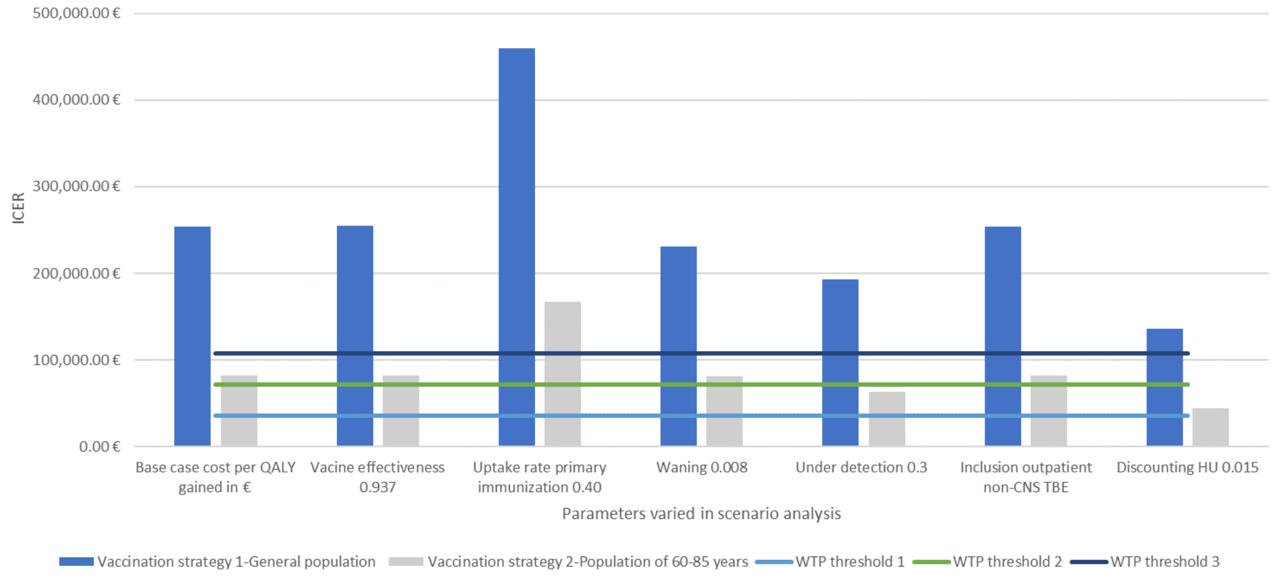

Table S1: Incidence thresholds for strategy 1 and 2 in different model scenarios using the real-world incidence

	Incidence threshold (WTP threshold 1)	Incidence threshold (WTP threshold 2)	Incidence threshold (WTP threshold 3)
**Strategy 1<br>General population (1-85 years)**			
**Base case**	3.98	2.19	1.51
**Vaccine effectiveness 0.937**	4.02	2.21	1.52
**Uptake rate primary immunization 0.40**	6.28	3.68	2.60
**Waning 0.008**	3.42	1.95	1.36
**Under ascertainment reported cases 0.3**	3.06	1.69	1.16
**Inclusion outpatient non-CNS TBE 0.15**	3.98	2.19	1.51
**Discounting HU 0.015**	2.34	1.24	0.84
**Strategy 2<br>60-85 years**			

**Base case**	1.49	0.77	0.52
**Vaccine effectiveness 0.937**	1.50	0.77	0.52
**Uptake rate primary immunization 0.40**	2.89	1.52	1.03
**Waning 0.008**	1.47	0.76	0.51
**Under ascertainment reported cases 0.3**	1.15	0.59	0.40
**Inclusion outpatient non-CNS TBE 0.15**	1.49	0.77	0.52
**Discounting HU 0.015**	0.82	0.41	0.28
